# Global Analysis of Transcriptome Responses and Gene Expression Profiles to Cold Stress of *Jatropha curcas* L.

**DOI:** 10.1371/journal.pone.0082817

**Published:** 2013-12-09

**Authors:** Haibo Wang, Zhurong Zou, Shasha Wang, Ming Gong

**Affiliations:** 1 School of Life Sciences, Engineering Research Center of Sustainable Development and Utilization of Biomass Energy, Ministry of Education, Key Laboratory of Biomass Energy and Environmental Biotechnology of Yunnan Province, Yunnan Normal University, Kunming, Yunnan, P. R. China; 2 College of Biological Resources and Environmental Science, Qujing Normal University, Qujing, Yunnan, P. R. China; Huazhong University of Science and Technology, China

## Abstract

**Background:**

*Jatropha curcas* L., also called the Physic nut, is an oil-rich shrub with multiple uses, including biodiesel production, and is currently exploited as a renewable energy resource in many countries. Nevertheless, because of its origin from the tropical MidAmerican zone, *J. curcas* confers an inherent but undesirable characteristic (low cold resistance) that may seriously restrict its large-scale popularization. This adaptive flaw can be genetically improved by elucidating the mechanisms underlying plant tolerance to cold temperatures. The newly developed Illumina Hiseq™ 2000 RNA-seq and Digital Gene Expression (DGE) are deep high-throughput approaches for gene expression analysis at the transcriptome level, using which we carefully investigated the gene expression profiles in response to cold stress to gain insight into the molecular mechanisms of cold response in *J. curcas*.

**Results:**

In total, 45,251 unigenes were obtained by assembly of clean data generated by RNA-seq analysis of the *J. curcas* transcriptome. A total of 33,363 and 912 complete or partial coding sequences (CDSs) were determined by protein database alignments and ESTScan prediction, respectively. Among these unigenes, more than 41.52% were involved in approximately 128 known metabolic or signaling pathways, and 4,185 were possibly associated with cold resistance. DGE analysis was used to assess the changes in gene expression when exposed to cold condition (12°C) for 12, 24, and 48 h. The results showed that 3,178 genes were significantly upregulated and 1,244 were downregulated under cold stress. These genes were then functionally annotated based on the transcriptome data from RNA-seq analysis.

**Conclusions:**

This study provides a global view of transcriptome response and gene expression profiling of *J. curcas* in response to cold stress. The results can help improve our current understanding of the mechanisms underlying plant cold resistance and favor the screening of crucial genes for genetically enhancing cold resistance in *J. curcas*.

## Introduction

To alleviate the energy crisis caused by increasing consumption of fossil fuels with limited reserves, more attention has shifted to the use of alternate renewable energy forms, such as biofuels, that are generally obtained from either carbohydrate- or oil-based feedstock or biomass. Oil-based feedstocks are mainly produced from the vegetable oils of agricultural plants, such as rapeseed, sunflower, soybean, and groundnut [1]. In many developing countries, the use of edible oils for biofuel purposes will certainly lead to a shortage in food and arable land. Therefore, non-edible oleaginous plants capable of growth in marginal lands with minimal inputs would be the best choice. *Jatropha curcas* has been suggested as a notable representative.

*J. curcas*, a woody shrub belonging to the Euphorbiaceae family, has been widely regarded as an excellent source of renewable biofuels, owing to its distinct features, including high seed oil content (30–50%) [2], fossil fuel-like oil composition (more than 75% unsaturated fatty acids) [3,4], and growth on degraded lands or wastelands in arid and semi-arid regions [1,5]. This plant species, primarily originating from Central America, has been recently introduced into many tropical and subtropical countries in Asia and Africa [3,6]. The generation period of *J. curcas* in tropical areas is only 6 months, with a high yield of 2000–4000 kg seeds/ha/year according to the study by Carels [7]. Nevertheless, possibly due to its tropical origin, *J. curcas* is extremely susceptible to cold, showing arrested growth and remarkable loss of seed yield [8], thus, restricting its extensive cultivation and distribution.

In recent years, numerous studies on *J. curcas* have mainly focused on its traits of commercial importance, such as seed yield, seed oil content, seed toxicity, synchronous maturity, and adaptations to biotic and abiotic stresses. Many functional genes associated with the above traits have been cloned [1], its genome of ~400 Mb [9,10] within 11 pairs of chromosomes [11-14] have been sequenced, transcriptome analyses of particular organs or developmental stages have been performed [15-17], methods for gene silencing have been established [18], and the first microsatellite- and single nucleotide polymorphism-based linkage maps have been generated [19]. However, with respect to the cold stress response, an unfavorable environmental factor for *J. curcas*, comprehensive analyses of the transcriptome response, and gene expression profile have not yet been made available, which is the exact aim of the current study.

By using the deep high-throughput sequencing approach Illumina Hiseq™ 2000 RNA-seq followed by Digital Gene Expression (DGE) analysis, we present, herein, a global transcriptome analysis and gene expression profile for *J. curcas* in response to cold exposure. From the deep sequenced transcriptome of a mixed sampling of *J. curcas* seedlings under normal condition and three cold treatments (12°C for 12h, 24h and 48h), a total of 45,251 unigenes were obtained and 33,848 could be annotated in nr protein database. Additional, individual DGE analysis revealed that 4185 genes were differentially expressed (upregulated or downregulated in the cold treatment versus the untreated control). The number of upregulated or downregulated genes changed gradually after 12 and 24 h of cold stress, whereas the upregulated number quickly increased within 24–48 h of cold treatments. These results are informative in clarifying the molecular mechanisms for the cold response and tolerance in *J. curcas*.

## Materials and Methods

### Seed germination and cold treatment of *J. curcas*

*J. curcas* seeds (from Yuanmou, Yunnan, China) were surface-sterilized in 1.5% CuSO_4_ for 30 min, rinsed thoroughly, and soaked in distilled water for 24 h. The imbibed seeds were sowed on six layers of wetted filter papers in trays and germinated in a climate chamber at 26°C in the dark for 5 d. Then, the geminated seeds were transferred to pots containing sterilized soil in a climate chamber at 26/20°C (day/night), with 75% relative humidity and a 16-h photoperiod, and sequentially grown for 14 d [20]. For the cold treatment, 2-week-old *J. curcas* seedlings were subjected to chilling at 12°C for 12, 24, and 48 h, respectively [21]. The leaves from the cold-treated and control seedlings (continually under normal growth conditions) were harvested, frozen in liquid nitrogen, and stored at -80°C until RNA extraction.

### Sample preparation for RNA-seq and de novo assembly of unigenes

Tissue samples from the control and three cold-treated *J. curcas* seedlings were mixed for total RNA extraction by a TRIzol reagent (Invitrogen). After a successive procedure consisting of mRNA enrichment by oligo(dT)-attached magnetic beads, mRNA fragmentation, cDNA synthesis with a random hexamer-primer, addition of the tailing A, and ligation of the adapters, the suitable fragments were purified and selected for sequencing templates by the Illumina Hiseq™ 2000 RNA-seq system. The clean data were obtained by discarding the low quality raw reads from the sequencing machines and used for *de novo* assembly of the unigenes by the Trinity program (Update Version: 2012-03-17) [22].

### DGE tag profiling

DGE tag profiling includes sample preparation by the Illumina Gene Expression Sample Prep Kit and sequencing by the Illumina Cluster Station and Hiseq™ 2000 RNA-seq system according to the manufacturer’s instructions. Briefly, for each of above four samples, the enriched mRNAs from 6 µg of the total RNA extraction were used to synthesize the double-strand cDNAs using the Oligo(dT) primer. After digestion with the restriction enzyme *Nla*III, which recognizes and cuts off the ↑CATG↓ sites, the cDNA fragments with intact Oligo(dT)-linked 3′ ends were purified by magnetic bead precipitation, ligated with Illumina adaptor 1 at the sticky 5′ ends, and digested by *Mme*I to generate cDNA tags with adaptor 1 at the 5′ end (the junction of adaptor 1 and the CATG site was designed as the obligatory part of the recognition site of *Mme*I, which cuts 17-bp downstream of the CATG site). Such tags were subsequently purified by removing the Oligo(dT)-linked fragments via magnetic bead precipitation and ligated with Illumina adaptor 2 at the sticky 3′ ends to achieve a library of 21 bp (CATG + 17 bp) cDNA tags with different Illumina adaptors at both ends. After 15 cycles of linear PCR amplification with the adaptor-specific primers GX1 and GX2, 105 bp fragments were purified by 6% PAGE, denatured, and fixed onto the Illumina Sequencing Chip (flow cell) by the Illumina Cluster Station for high-throughput sequencing by using Illumina Hiseq^TM^ 2000 RNA-seq system. By discarding a few low-quality items and trimming the adaptor sequences from the raw sequencing reads, the clean data of 21 bp tags with CATG at the 5′ end were acquired.

### Aligning DGE tags to reference transcriptome data generated by RNA-seq

Sequence quality assessment for the DGE tags was carried out according to the Illumina pipeline, including the distribution of the total tags, distinct tags, clean tag copy number, and saturation of sequencing. All clean tags were mapped to the reference transcriptome generated by RNA-seq. Firstly, the CATG sites were scrutinized on each mRNA from the transcriptome to construct the reference tag database with 21 bp (4 bp ‘CATG’ + 17 bp), to which all clean DGE tags were then aligned, with only a 1-bp mismatch of tolerance. The number of unambiguous clean tags for each gene was calculated and then normalized to the number of transcripts per million clean tags (TPM) [23,24] to obtain the relative gene expression level.

### GO functional classification of differentially expressed genes

All differentially expressed genes were functionally categorized by mapping them to the Gene Ontology database using the map2slim program. The percentage of each resultant functional category was calculated with regular spreadsheet analysis programs. The *p* value for significance of a particular GO category was calculated using the hypergeometric test, and the main biological functions of the corresponding terms were identified.

### Identification of differentially expressed genes associated with cold resistance

By using the alignment data of the DGE tags against the reference transcriptome, rigorous algorithms were developed to identify differentially expressed genes associated with cold resistance between any of the two aforementioned samples according to the descriptions of Audic [25]. Furthermore, the *p* value and false discovery rate (FDR), two-feature parameters in multiple tests, were also introduced [26]. Assuming that R differentially expressed genes have been selected, in which S genes actually showed differential expression, while the other V genes were false positives, the error ratio Q = V/R must remain below a cutoff (1%). In our study, the absolute value of the Ratio within the range of 1~2 and p ≤ 0.01 & FDR ≤ 0.001, and the absolute value of the Ratio ≥ 2 (namely absolute value of the log_2_Ratio ≥ 1) were used as the thresholds to assess the ‘difference’ and ‘significant difference’ of gene expression between two samples, respectively. 

## Results

### Statistics of RNA-seq transcriptome data for *J. curcas*

Using Illumina Hiseq™ 2000 RNA-seq, approximately 55,112,142 clean reads (4,960,092,780 nt total), with an average G + C content of 42.16%, were generated from a mixed transcriptome of *J. curcas* seedlings. Through *de novo* assembly, a total of 45,251 unigenes, with an average length of 789 nt, were obtained, and 35,791 (79.09%) were successfully annotated into different databases, such as nr, nt, Swiss-Prot, GO, COG, and KEGG. Additional BlastX and ESTScan analyses revealed that 34,274 unigenes had reliable CDSs (33,362 derived from the database alignment by BlastX and 912 from the ESTScan prediction). All annotated and CDS-containing unigenes were used to constitute a reference database for DGE analysis.

### DGE analysis for *J. curcas* under cold stress

DGE analysis [27] was performed to identify gene expression changes in *J. curcas* seedlings caused by cold exposure. A total of 4.95, 4.68, 4.87, and 4.95 million raw tags of the mRNAs extracted from the leaves after 12°C cold treatments for 0 (control), 12, 24, and 48 h were obtained, and approximately 4.81, 4.49, 4.70, and 4.79 million high-quality, non-redundant clean tags were identified after filtering the dirty tags, respectively. Tag annotation was carried out by tag mapping to a reference database (i.e., the aforementioned RNA-seq-based transcriptome; 45,251 reference unigenes, including 36,708 unigenes with CATG sites, and 113,216 reference tags were generated). Accordingly, the results showed that 69.86%, 66.76%, 65.26%, and 71.00% of all distinct clean tags from the above four cold treatments were individually mapped to the reference tag database (sense or antisense). Meanwhile, the relative expression level for each *J. curcas* unigene with a DGE tag match in each cold treatment was calculated and used for identification of differentially expressed genes associated with cold stress.

### Identification of differentially expressed unigenes of *J. curcas* under cold stress

**Figure 1:**
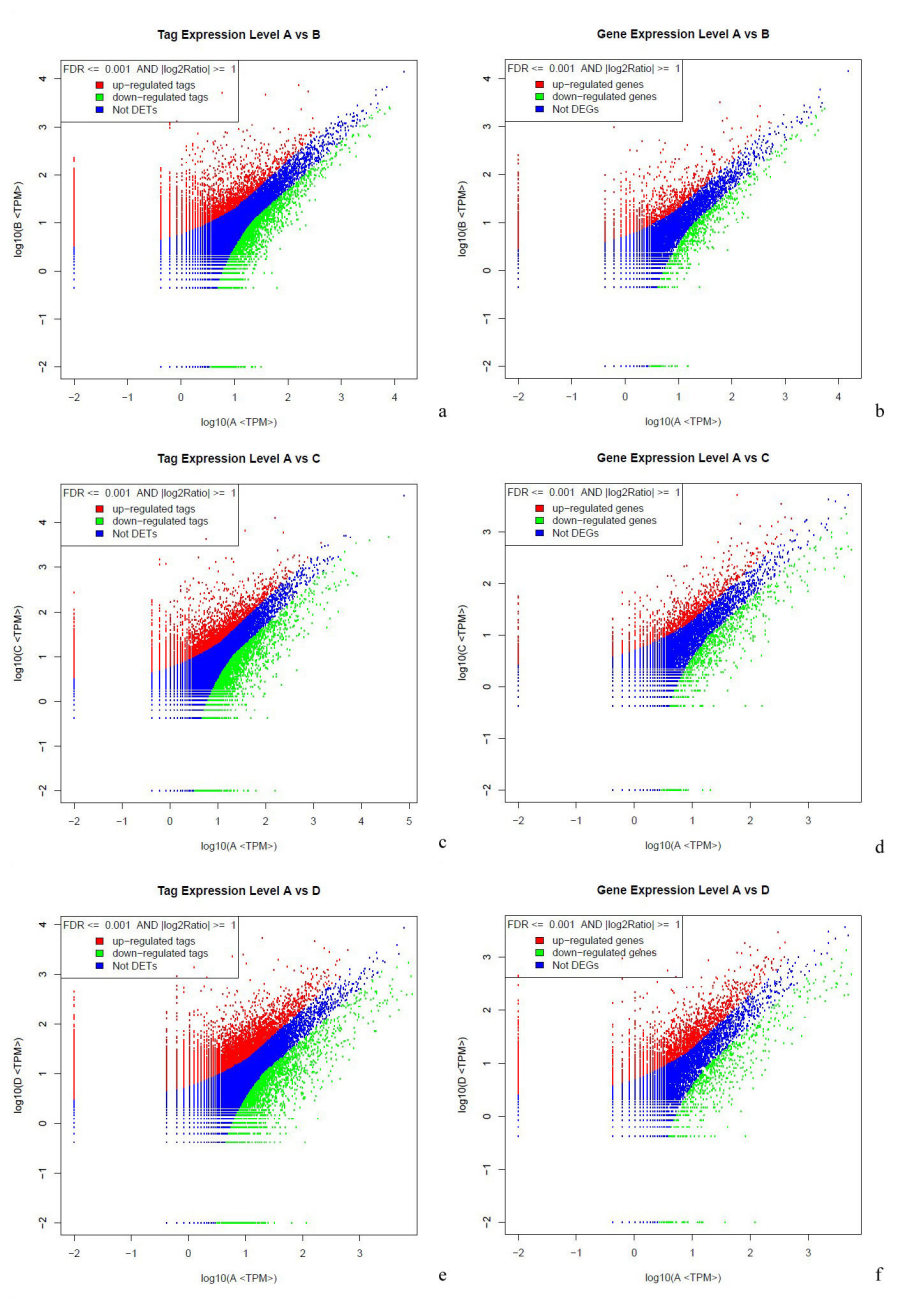
Differential expression analysis of tags and unigenes by DGE. The expression level for each tag and unigene is shown in the volcano plots (a, c, e) and (b, d, f) after 12 (a, b), 24 (c, d), and 48 h (e, f) of cold stress. ‘Not DETs’ indicates ‘not detected expression tags’ and ‘Not DEGs’ indicates ‘not detected expression genes’. The horizontal line indicates the log_10_ of transcripts per million of the control (A) and the vertical line indicates the log_10_ of transcripts per million of 12 (B), 24 (C), and 48 h (D) of treatment at 12°C. The criteria for screening differentially expressed individuals are based on FDR ≤ 0.001 and the absolute value of log_2_Ratio ≥ 1.

**Figure 2:**
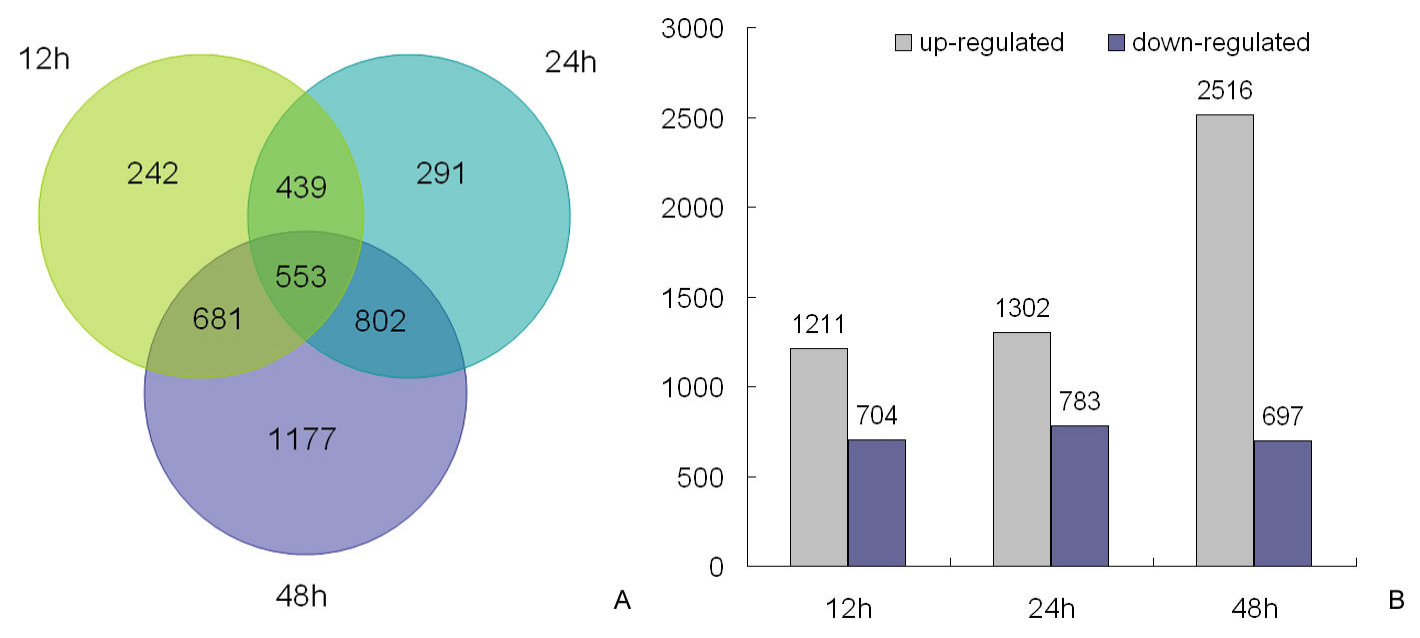
The differentially expressed genes of *Jatropha curcas* after cold exposure for schemed time points. A: Venn diagram indicating the total number of differentially expressed genes after 12, 24, and 48 h of 12°C (≥2-fold change in expression). B: Classified number of upregulated and downregulated genes for each time point of the three cold treatments.

 Based on the criteria for screening differentially expressed genes (Figure 1), a total of 4185 *J. curcas* genes from the reference transcriptome (45,251 unigenes) were found to be differentially expressed after three cold treatments (12°C for 12, 24, and 48 h) in comparison to the untreated control (constant exposure at 26°C ). Therein, 1915, 2085, and 3213 genes corresponded to 12, 24, and 48 h of cold exposure, respectively, and 553 genes were common to each treatment (Figure 2A). 

In detail, 1211 genes of *J. curcas* underwent rapid upregulation at 12 h of cold exposure, which concomitantly occurred with the downregulation of 704 genes. After 24 h of cold stress, 1302 and 783 genes were upregulated and downregulated for expression, respectively. In contrast, the number of upregulated genes increased drastically to 2516 after 48 h of cold stress, whereas the number of downregulated genes changed only slightly (Figure 2B). This scenario implies that the global gene activation of *J. curcas* in response to cold stress occurs slowly during the early stages, but increases with duration up until a certain time point. Nevertheless, the number of downregulated genes did not appear to be affected by the duration of cold exposure.

**Figure 3:**
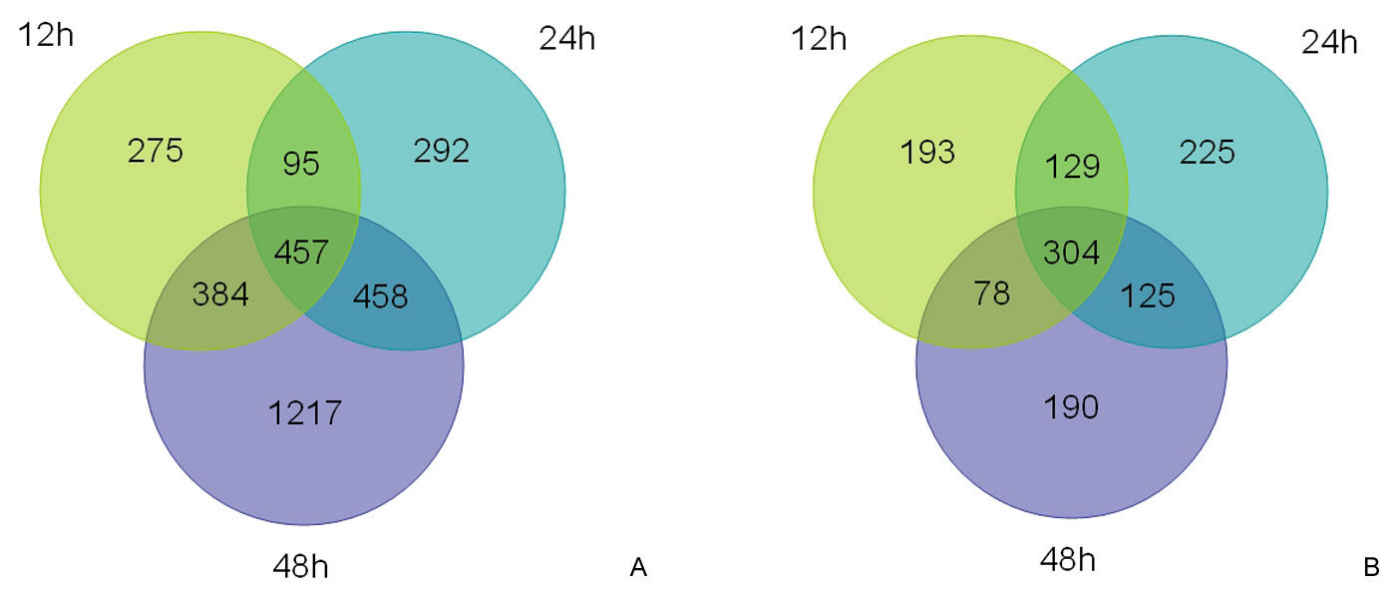
Venn diagrams showing differentially expressed *Jatropha curcas* genes after cold exposure for three time points. A: upregulated genes. B: downregulated genes.

Additionally, an overall statistical analysis classified the cold-responsive *J. curcas* genes that were differentially expressed, with ~3178 and 1244 genes were upregulated and downregulated, respectively (some genes were inconsistently upregulated or downregulated for each of the three time points). Venn diagrams were constructed to further specify these genes for each time point of cold exposure (Figure 3). In total, 275 and 292 genes of *J. curcas* were particularly upregulated after 12 and 24 h, respectively, whereas a drastic increase in number (1217) was induced only after 48 h of cold treatment. Notably, 457 (14.4% of 3178 upregulated genes) were common among all three time points, implying their considerable importance in the cold stress response (Figure 3A). In parallel, it was also observed that 193,225, and 190 genes were uniquely repressed after 12, 24, and 48 h of cold treatments, respectively, which accompanied the common downregulation of 304 (24.4% of 1244) genes (Figure 3B).

Of the 457 common genes of *J. curcas* upregulated in all three cold treatments, candidates were identified for their involvement in encoding cytochrome P450 (e.g., Unigene3466_JC-CK_1A), photosynthesis (e.g., Unigene536_JC-CK_1A) and ubiquinone (e.g., Unigene2514_JC-CK_1A), which are involved in electron transportation and redox equilibrium. Moreover, genes encoding β-amylase (e.g., Unigene804_JC-CK_1A), gibberellin oxidase (e.g., Unigene3452_JC-CK_1A), cation-transporting ATPase (e.g., Unigene1535_JC-CK_1A), and non-specific lipid-transfer protein (e.g., Unigene869_JC-CK_1A; belonging to the hydrolase and micromolecule transporter family) were strongly upregulated, thus suggesting their participation in the cold response and resistance in plants, possibly by decreasing cellular osmotic potential. In addition, constantly cold-induced genes also include those encoding DNA- or RNA-binding proteins (e.g., Unigene5022_JC-CK_1A), transcription factors (e.g., Unigene2363_JC-CK_1A and Unigene2304_JC-CK_1A for AP2/ERF and ABC transcription factors, respectively), signal transduction proteins (e.g., Unigene2287_JC-CK_1A and Unigene2573_JC-CK_1A for auxin- and ethylene-responsive proteins, respectively), stress proteins (e.g., CL815.Contig1_JC-CK_1A for late embryo abundant-5), Ser/Thr-type kinases (e.g., Unigene3411_JC-CK_1A), and more.

### Functional classification of cold-affected *J. curcas* genes by Gene Ontology analysis

**Figure 4:**
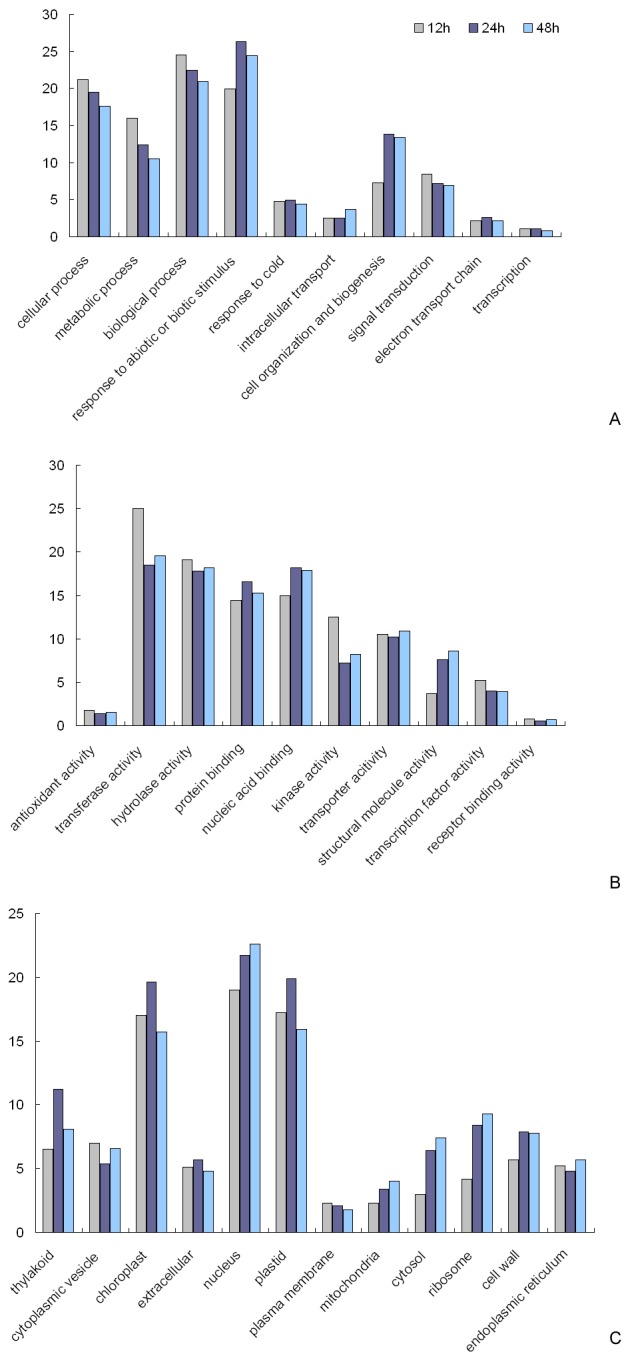
Gene Ontology classification of differentially expressed genes of *Jatropha curcas*. A: Biological Process. B: Molecular Function. C: Cellular Component. The vertical line indicates the percentage of corresponding genes.

To investigate the identity of *J. curcas* genes differentially expressed under cold stress, functional categorization was carried out by Gene Ontology (GO) analysis using the map2slim program. Finally, all 4185 differentially expressed genes revealed by DEG analysis were functionally assigned to the relevant terms in three categories (‘Biological Process’, ‘Molecular Function’, and ‘Cellular Component’) of the GO database using the hypergeometric test (Figure 4). 

In the category of ‘Biological Process’, the GO-terms ‘response to abiotic or biotic stimulus’ and ‘cell organization and biogenesis’ were significantly overrepresented, especially after 24 h of cold exposure. Additionally, the percentage of *J. curcas* genes correlated with the term ‘response to cold’ was notable and identical in all three cold treatments. Other terms such as ‘cellular process’, ‘metabolic process’, ‘biological process’, and ‘signal transduction’ were also dominant, despite a decrease in occurrence with an increase in duration of cold exposure. These data indicated the existence of signaling processes and an inhibition of cellular activity in *J. curcas* under cold stress (Figure 4A). 

When looking into the category of ‘Molecular Function’, several GO terms were overrepresented, such as ‘transferase activity’, ‘kinase activity’, ‘hydrolase activity’, ‘transcription factor activity’, ‘protein binding’, ‘nucleic acid binding’, and ‘structural molecule activity’. The former four terms were clearly identified with a decrease in the percentage of involved unigenes under prolonged period (>12 h) of cold exposure, while the latter three terms, in contrast, were associated with an increase. These data, overall, suggest a general repression of metabolic activities under cold stress. Nevertheless, cold-induced metabolic pathway changes include a remarkable increase depending on the binding-type transcription factor, thus maintaining the basic cold-resistant metabolic efficiency and facilitating cell membrane or cell wall remodeling. In addition, a majority of transcription factors might be evoked in response to cold stimuli as early as 12 h but become somewhat silenced under prolonged cold stress, thus implying that cold signaling in *J. curcas* is likely an early event (Figure 4B). 

Meanwhile, in the category of ‘Cellular Component’, an increased percentage of *J. curcas* genes corresponding to the terms ‘cytosol’, ‘mitochondria’, ‘thylakoid’, and ‘cell wall’ was found under prolonged cold exposure (>12 h), which hints that the regulation of osmotic potential, energy balance, and photosynthesis efficiency may play an important role in cold stress. In addition, the GO terms ‘nucleus’ and ‘ribosome’, which are indicative of the vigorousness in transcription and translation, were also overrepresented and exhibited an additional gradual increase in gene participation during prolonged periods of cold stress (Figure 4C). On the basis of these findings, we assumed that the comprehensive intracellular rearrangement might be occurred during cold stress and enable a reshuffling of resources from cell membrane and cell wall structural modification in the outer parts of the cell towards osmotic potential and cold-resistant substance increasing via energy metabolism balance and transcription factor-dependent metabolic pathway alteration in the inner cell [28].

### Starch catabolism-related genes

**Figure 5:**
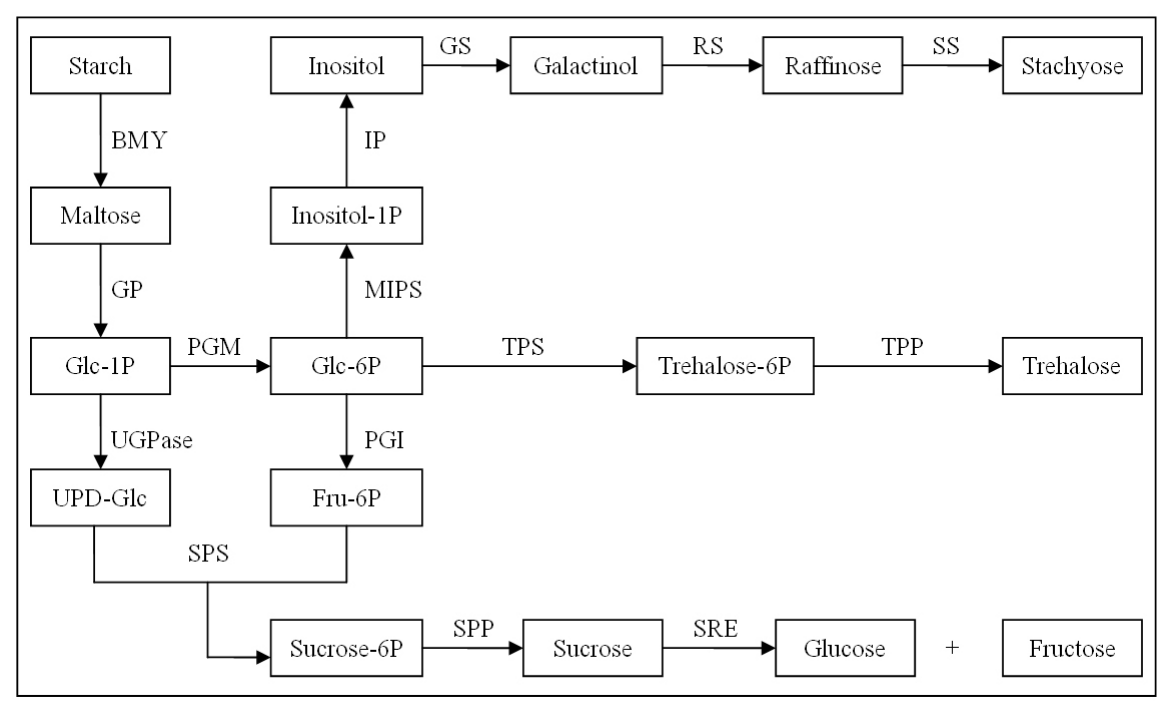
Simplified model of starch catabolism pathway.

**Figure 6:**
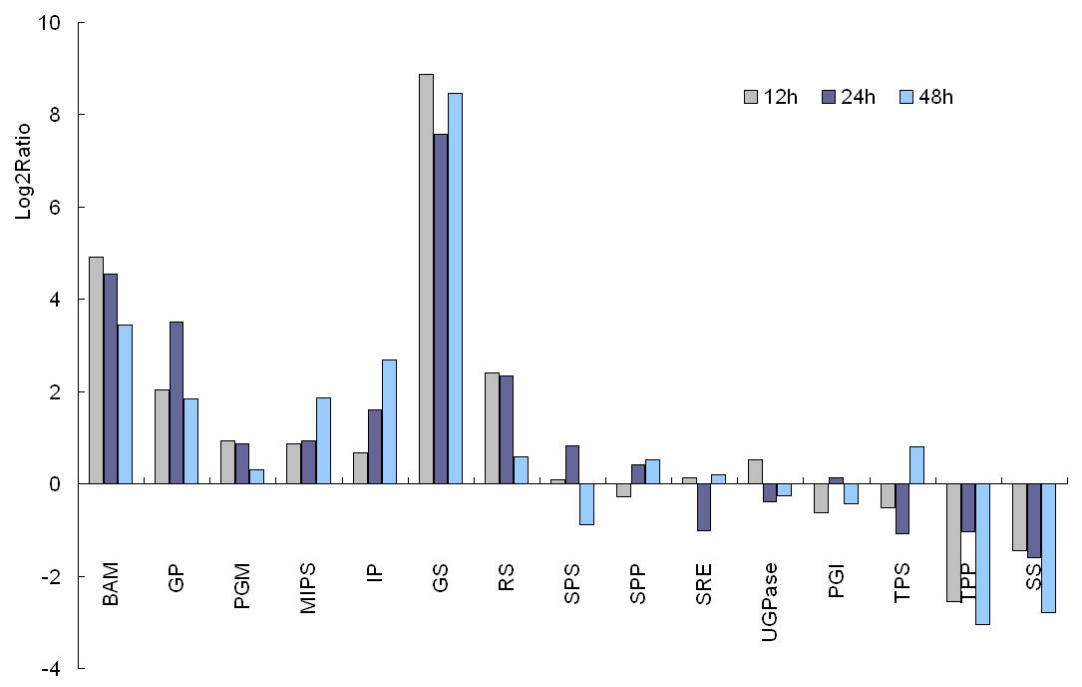
Expressions of enzymes relevant to starch metabolism after 12, 24, and 48 h at 12°C.

**Figure 7:**
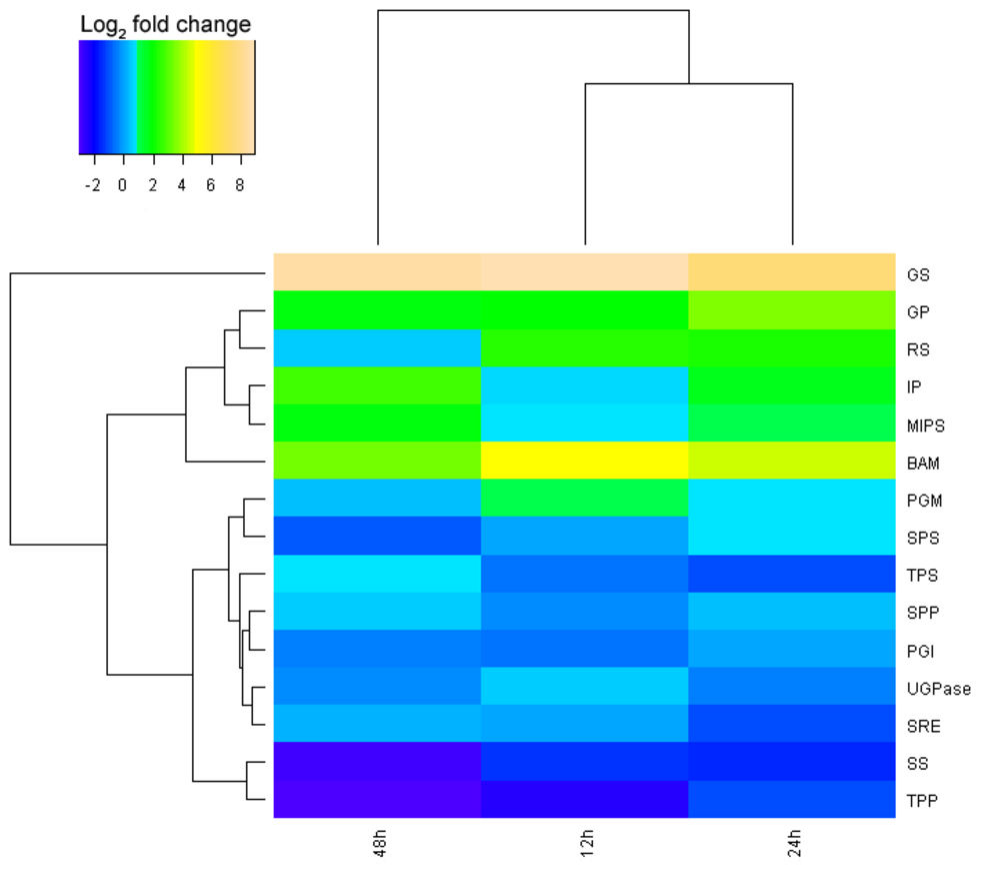
Clustering analysis of differential gene expression pattern relevant to starch catabolism.

The role of sugar accumulation in the development of plant cold tolerance has been well documented [29]. As a type of osmolyte, sugar can not only change the cellular osmotic potential to protect the plasma membrane from cold damage but can also favor reactive oxygen species (ROS) scavenging [30]. We reconstructed the primary pathway of starch catabolism [31] for clarifying the gene expression levels of major enzymes (Figures 5, 6, and 7). As indicated in Table S1 and Figure 6, the dramatically upregulated enzymes involved in starch turnover (starch to Glu-1P) were β-amylase and glucan phosphorylase, each displayed a 3.61-fold increase at minimum after all three time points (12 h, 24 h, 48 h) of exposure at 12°C. Surprisingly, under all three cold treatments, a moderate increase in gene expression occurred for the enzymes related to the biosynthesis of inositol (e.g., phosphoglucomutase, myo-inositol 1-phosphate synthase, and myo inositol monophosphatase), and a remarkable increase in those of galactinol and raffinose (467.88- and 5.31-fold increase for galactinol synthase and raffinose synthase, respectively, after 12 h cold exposure). In contrast, two key enzymes for sucrose production (sucrose phosphate synthase and sucrose phosphate monophosphatase) were slightly and simultaneously upregulated only with 24 h of cold exposure; the other two correlated enzymes (uridine diphosphate glucose [UDPG] pyrophosphorylase and glucose-6-phosphate isomerase) were slightly downregulated for gene expression. Meanwhile, the expression levels of crucial enzymes (trehalose-6-phosphate synthase, trehalose phosphate phosphatase, and stachyose synthase) for synthesizing other soluble sugars (e.g., trehalose and stachyose) decreased under cold stress. These results suggest that the sugars (analogue) galactinol, raffinose, and/or inositol, instead of the better known sucrose or trehalose, might play a major role in the cold resistance of *J. curcas.*

### Identification of ABA-independent signal transduction pathway genes

**Figure 8:**
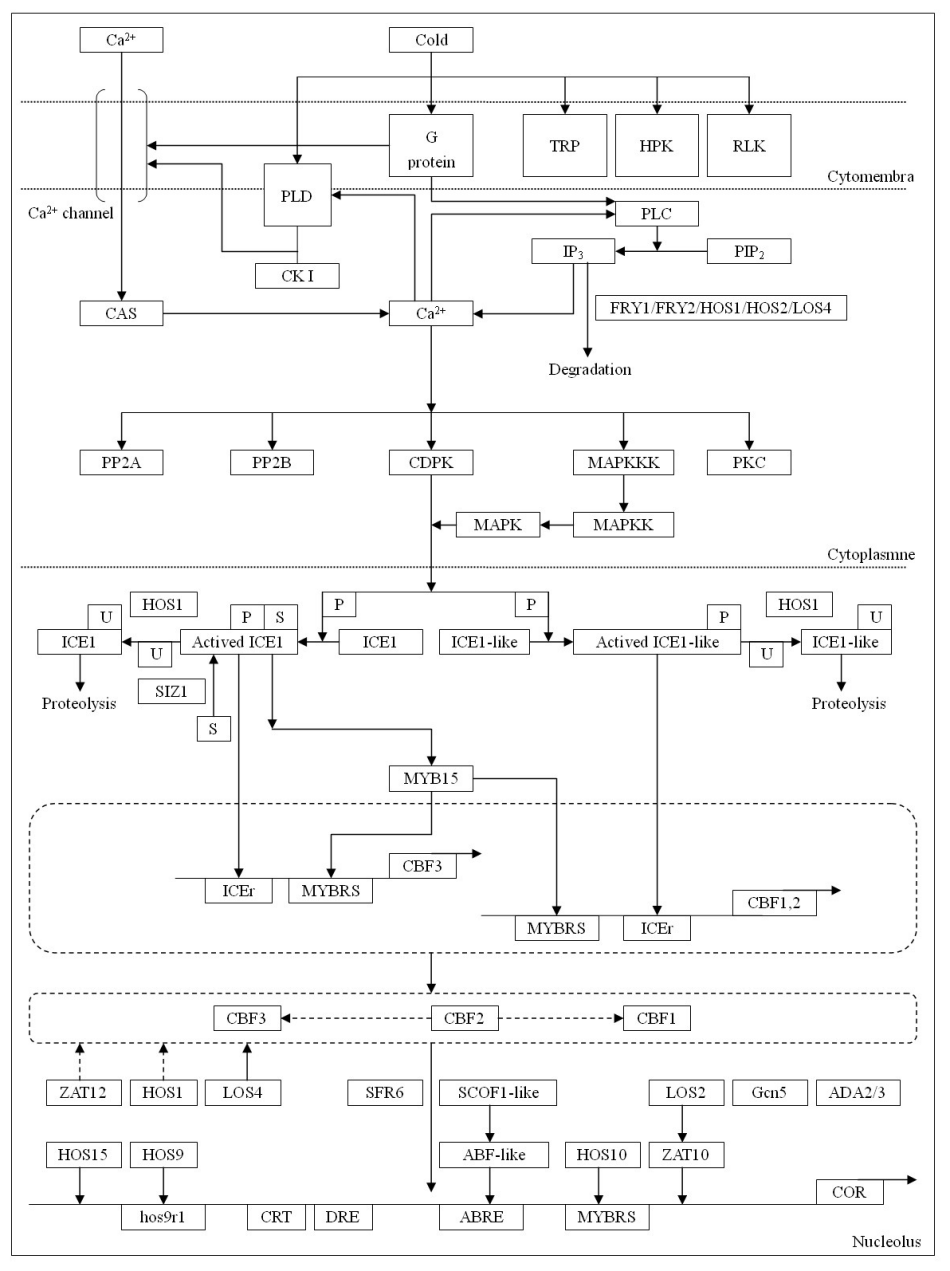
Putative ABA-independent signal transduction pathway. Putative ABA-independent signal transduction pathway was constructed based on reference 31. HPK: two-component histidine kinase; RLK: receptor protein kinase; TRP: transmembrane responsive protein; PLC: phospholipase C; PLD: phospholipase D; CK I: casein kinase I; PIP_2_: phosphatidylinositol 4,5-biphosphate; IP_3_: inositol 1,4,5-trisphosphate; CDPK: calcium dependent protein kinase; PP2A: protein phosphatase 2A; PP2B: protein phosphatase 2B; PKC: protein kinase C; MAPK: mitogen-activated protein kinase; MAPKK: MAPK-kinase; MAPKKK: MAPKK kinase; ICEr: ICE recognition motifs; MYBRS: MYB recognition sequence; Hos9r1: Hos9 recognition motif 1; U: ubiquitin protein; S: SUMO protein; P: phosphoryl group; HOS1: RING E3 ligase; HOS10: R2R3-type MYB transcription factor; HOS15: WD40-repeat protein; FRY1/2: inositol polyphosphate 1-phosphatase; SIZ1: SUMO E3 ligase; SFR6: sensitive to freezing 6; Gcn5: histone acetyltransferase enzyme; ADA2/3: transcriptional adaptor protein 2/3.

**Figure 9:**
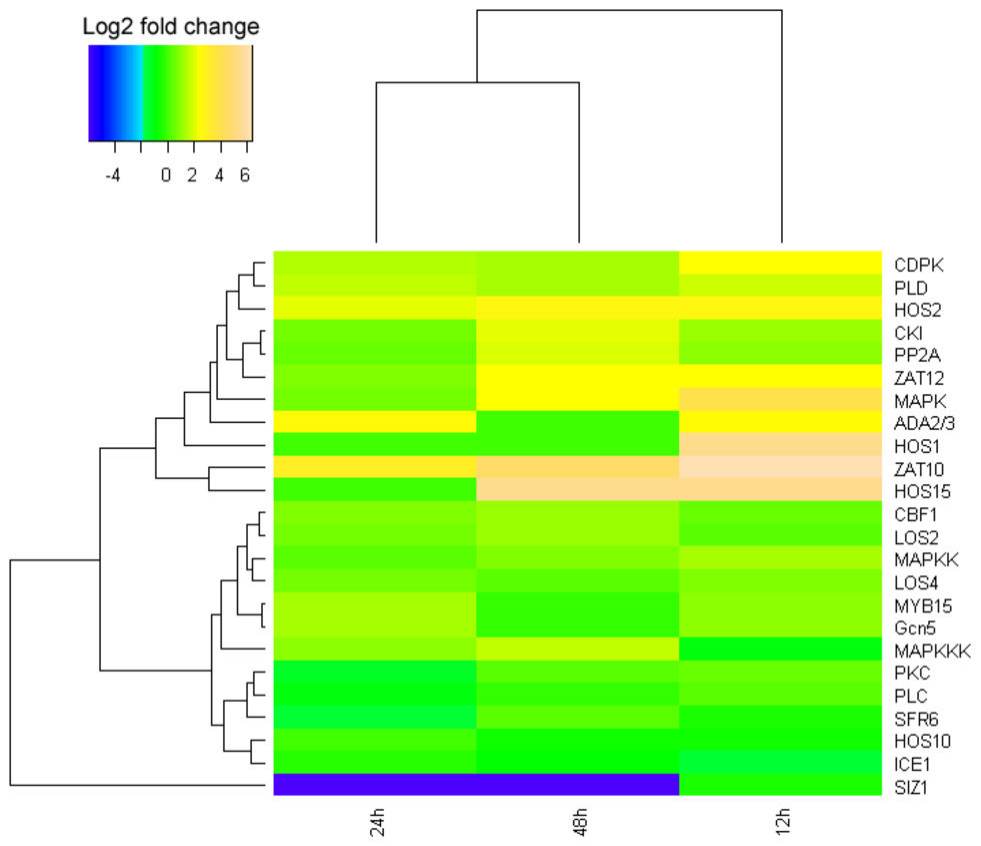
Clustering analysis of differential gene expression pattern relevant to ABA-independent signal transduction pathway.

The ABA-independent signal transduction pathway has been well clarified for the cold response and resistance in plants, especially the C-repeat binding factor (CBF) pathway [32]. We reconstructed the ABA-independent signal transduction pathway for identifying differentially expressed genes and related transcription factors (Figures 8 and 9) [31]. The CBF pathway typically displays an immediate increase in the concentration of cytosolic calcium ([Ca^2+^]cyt), probably mediated by IP_3_ through the activity of phospholipase C (PLC) [33], which is clearly an event of the signaling cascade leading to the induction of cold-responsive gene expression. This suggests that PLC-released IP_3_ might be involved in the calcium response during cold stress [34]. In addition, some of the transcription factors, such as HOS1, HOS2, LOS4, and FRY1/2 (encoding inositol polyphosphate 1-phosphatase), which participate in the proteolysis of IP_3_, play a crucial role in balancing IP_3_ concentrations. Simultaneously, phospholipase Dδ (PLDδ), anchored in the plasma membrane, can cause a rearrangement in the conformation of the cytoskeleton, and thus activate and open the Ca^2+^ channel [35,36]. In our DGE analysis, PLDδ was upregulated for expression (3.43-fold after 12 h at 12°C) more highly than PLC (1.21-fold after 12 h at 12°C), suggesting cold-induced enrichment of cytosolic Ca^2+^ mainly depends on the activity of PLD in *J. curcas*.

The data showed that some proteins were phosphorylated, whereas others were dephosphorylated during cold exposure. These changes in phosphorylation status can affect gene induction. For example, calmodulin, a calcium-binding protein, can activate many kinases responsible for the signaling cascade, including calcium-dependent protein kinases (CDPK), casein kinase I (CK I), protein kinase C (PKC), and mitogen-activated kinases (MAPK). All of these kinases were somewhat upregulated for expression during early and/or prolonged periods of cold exposure (Table S1), especially MAPK (15.14-fold after 12 h at 12°C). MAPK was activated by phosphorylation under the action of MAPK-kinases (MAPKK), which are also phosphorylated by MAPKK-kinases (MAPKKK). In our DGE analysis, protein phosphatase 2A (PP2A), a main phosphatase in plants, was induced by low temperature in *J. curcas*. A 3.78-fold upregulation after 48 h at 12°C implies that PP2A is involved in the regulation of the CBF pathway in *J. curcas*. Another PP2C-type phosphatase, which acts as an MAPK phosphatase, has been found in *Arabidopsis* but was not detected in *J. curcas*. Thus, the balance of MAPK phosphorylation is dependent on other ways in *J. curcas*.

Transcription factors are important for cold signaling and tolerance by modulating the expression of correlated functional genes [14]. In the ABA-independent cold transduction pathway, CBF and its upstream regulator, ICE, are the main transcription factors with a considerable number of studies up to now [32]. The CBF unigenes in *J. curcas*, as well as those encoding other related regulatory factors, such as HOS1 [37,38], HOS15 [39], LOS2 [40], LOS4 [41], ZAT10 [42], and ZAT12 [43], were definitively shown to be upregulated in our DGE analysis (Table S1). Among them, HOS1 (one ring finger protein) may act as an upstream and negative regulator of CBF. It may also participate in the ubiquitination of ICE1 and ICE-like [44], and the degradation of IP_3_, a main intracellular and extracellular signaling molecule. During 12°C cold exposure for 24 h and 48 h, expression of HOS1 was not found (Table S1), implying that its absence might increase the concentration of ICE and, thus, enhance CBF expression [37,38]. MYB15, a R2R3-MYB protein, could physically interact with ICE1 and act as a negative regulator of CBF expression by binding to the MYB recognition sites of the CBFs. DGE analysis showed that it is upregulated after 12 and 24 h at 12°C but downregulated after 48 h, thus implying that depression for CBF expression gradually decreased following cold treatment. Of the two ZAT factors identified, both were upregulated. ZAT10, as a zinc finger protein and a negative regulator of CBF genes, was upregulated remarkably (e.g., 81.57-fold after 12 h at 12°C). However, its activity was antagonized by an enolase-like transcription factor LOS2 that was also upregulated for expression concomitantly, which finally activated the CBF pathway to increase plant cold resistance. (e.g., 2.35-fold after 48 h of cold treatment) [40] (Figure 7 and Table S1). ZAT12, which contains the ICEr3- and ICEr4-binding motifs in the promoter, is a protein with zinc-finger and EAR domains and, in response to cold stress, can negatively influence the CBF pathway (5.03-fold after 12 h at 12°C) [43]. Conversely, LOS4, a DEAD-box RNA helicase, promoted the expression of CBF1-3, showing a 1.84-fold after 12 h of cold treatment [41]. These data show that the expression of CBFs is not only regulated by ICE but also requires the participation of many auxiliary factors.

### Novel genes induced by cold stress in *J. curcas*

**Figure 10:**
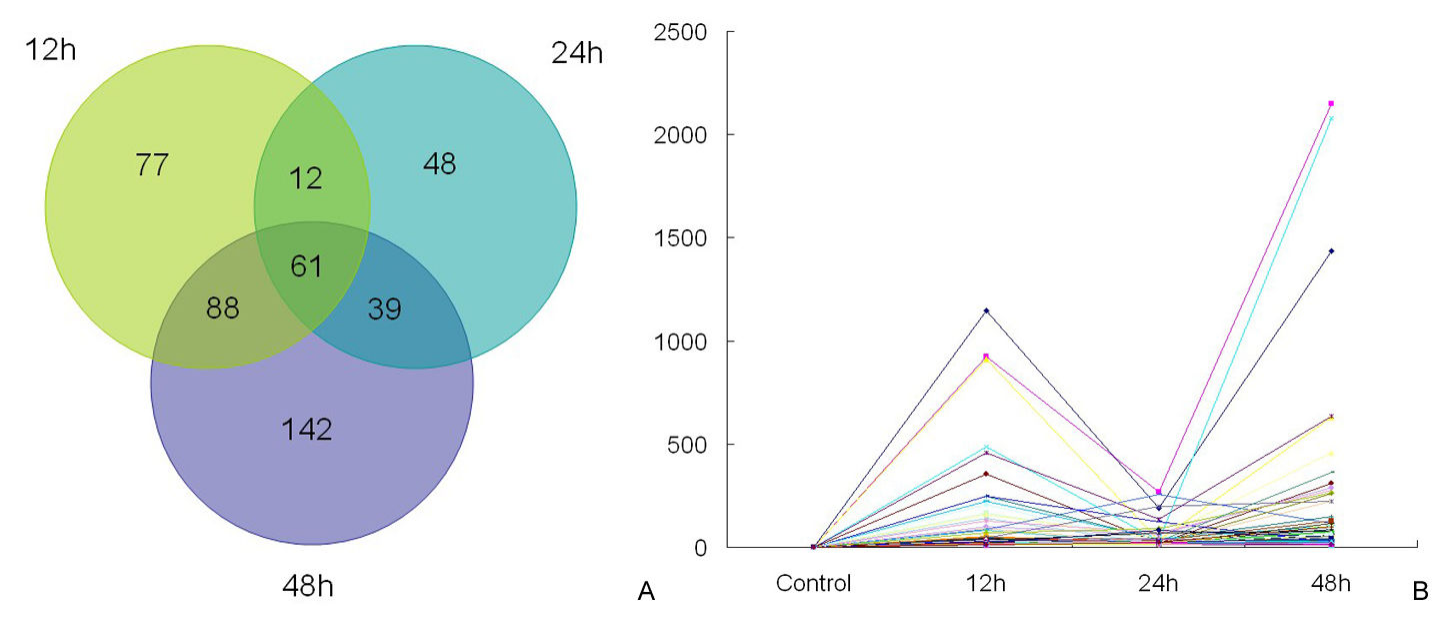
Number and expression model of novel *Jatropha curcas* genes after cold exposure for schemed periods. A: Venn diagram indicating the total number of novel, expressed genes after 12, 24, and 48 h of 12°C. B: Expression model of common, novel genes at three time points. The vertical line indicates the raw expression of novel genes in each sample.

To further illustrate the novel, expressed genes at all three time points, we constructed a Venn diagram (Figure 10A and Table S2). Therein, 238, 160, and 330 novel genes were individually induced after 12, 24, and 48 h of cold treatment, respectively. Moreover, we identified 61 genes that were commonly expressed at all three time points. Among them, the five most highly expressed novel genes possibly encoding cucumber peeling cupredoxin-like protein (Unigene739_JC-CK_1A, 2148 raw expression level, same below), major allergen Pru ar-like protein (Unigene790_JC-CK_1A, 2080), chaperone protein DNAj (CL884.Contig1_JC-CK_1A, 1435), receptor protein kinase CLAVATA1 precursor-like protein (Unigene2608_JC-CK_1A, 637), and conserved hypothetical protein (Unigene1239_JC-CK_1A, 632), depending on nr annotation (Figure 10B and Table S2). A total of 77, 48, and 142 genes were uniquely expressed after 12, 24, and 48 h, respectively. 

Among the 61 commonly expressed novel genes, most of them increased notably at 12 and 48 h of cold exposure, especially after 48 h, whereas expression decreased at 24 h (Figure 10B). Four of the commonly expressed genes related to transferase (benzoate carboxyl methyltransferase Unigene6237_JC-CK_1A; methionine S-methyltransferase Unigene16350_JC-CK_1A; glycosyl transferase Unigene3916_JC-CK_1A; and transferase Unigene1986_JC-CK_1A) were a part of the network of covalent modification, which activate or inhibit enzyme activity. The annotation revealed that assistant proteins were abundant in the novel, expressed genes and belonged to several families of DNA-binding proteins (e.g., RAB6-interacting protein Unigene6191_JC-CK_1A; MADS-box protein Unigene19444_JC-CK_1A; DNA-binding proteins Unigene10927_JC-CK_1A and Unigene2558_JC-CK_1A; chaperone protein DNAj CL884.Contig1_JC-CK_1A; and ethylene-responsive element-binding protein Unigene2573_JC-CK_1A), receptor proteins (e.g., receptor protein kinase CLAVATA1 Unigene2608_JC-CK_1A and ethylene receptor CL5164.Contig1_JC-CK_1A), transporters (e.g., amino acid transporter Unigene7216_JC-CK_1A and abc-transporter Unigene2594_JC-CK_1A), suggesting signal transduction plays a core role in *J. curcas* cold tolerance. In addition, there were several novel genes coding for cytochrome P450 (Unigene1857_JC-CK_1A and Unigene3466_JC-CK_1A), reticuline oxidase (CL210.Contig1_JC-CK_1A), rotenone-insensitive NADH-ubiquinone oxidoreductase (CL4339.Contig1_JC-CK_1A), gibberellin 2-oxidase (Unigene2535_JC-CK_1A), and cucumber peeling cupredoxin-like (Unigene739_JC-CK_1A), which are known to be involved in oxidation–reduction equilibrium during cold stress.

## Discussion

*J. curcas* is a sustainable energy plant with great potential for biodiesel production but is severely restricted by low temperature for its distribution and cultivation. During the past few years, the effects of cold exposure on *J. curcas* physiology and development, such as a reduced percentage of seed germination and increased activity of the antioxidant defense system [21], have been reported in detail. Nevertheless, the cold-induced molecular changes remain largely unknown. For global gene expression analysis, the newly developed Illumina RNA-seq and DGE approaches have dramatically expanded insights into transcriptome and functional gene expression, with particular advantages that make possible the detection of alternative splicing events, low-abundance transcripts, and novel genes [45]. By using the above techniques, we provided, herein, the most up to date and comprehensive profiling of cold-correlated *J. curcas* genes (4185 members in total, including novel genes) and their differential changes in expression (Figure 2, Table S1), which may greatly depend on the current understanding of the cold-resistance mechanism in *J. curcas* and other plant species. Current studies have already showed that plant cold tolerance can be ascribed to several cellular behaviors, such as changing membrane fluidity, increasing the concentration of various osmoprotectants, and improving the activity of antioxidation through cold signaling, and ultimately, a differential pattern of gene expression [31].

### Fatty acid unsaturated genes

Cold exposure can induce distinct changes in membrane composition, especially with a notable increase in the unsaturation degree of phospholipids [46-48]. The unsaturation level of lipids has been proposed to play a role in increasing membrane fluidity and preventing cold-induced membrane damage (e.g., expansion-induced lysis). After cold exposure, the proportion of di- and tri-unsaturated acyl-chains, such as 18:1/18:3, 18:2/18:2, and 18:2/18:3, increased in both phosphatidylcholine and phosphatidylethanolamine, and the proportion of mono-unsaturated species, such as 18:0/18:3 and 16:0/18:3, decreased [49]. In the current study, the key genes related to fatty acid unsaturation were remarkably regulated after 24 h of cold stress. Therein, genes encoding △^9^-stearoyl-ACP desaturase (SAD) and △^12^-Fatty acid desaturase (*FAD2*) were upregulated and were responsible for the conversion of stearoyl-ACP (18:0) to oleoyl-ACP(18:1) and oleoyl-ACP(18:1) to linoleoyl-ACP(18:2), respectively. As polyunsaturated fatty acids, trienoic fatty acids are thought to prevent the transition from the liquid crystalline phase to the gel phase in chloroplast membranes and recovery from photo-inhibition in thylakoid membranes of *Arabidopsis*. FAD8, a chloroplast ω-3 desaturase involved in trienoic acid synthesis, markedly increases in *Arabidopsis* plants grown at low temperatures. In our DGE analysis, one ω-3-fatty acid desaturase was also upregulated for its expression (Table S1), which was consistent with an increase in the content of trienoic acid under cold stress.

### Antioxidative enzyme and antioxidants regenerating related genes

Cold exposure often induces oxidative stress in plants. To counterbalance the excessive ROS formed in the chloroplast (in an earlier section), mitochondria (in the UQ/UQH_2_ cycle), peroxisome, or cytosol, the plant interior antioxidative system, consisting of the scavenging enzymes and antioxidants, is activated. In this study, cold-inducible peroxidase (POD) and Zn-Superoxide dismutase (SOD) [50,51], which directly scavenge for excessive ROS, showed 48.50- and 1.61-fold increases in expression after 12 h of exposure to 12°C, respectively (Table S1). The results indicate that tolerance to chilling in *J. curcas* is associated with the enhanced capacity of the antioxidative enzyme under cold stress [31]. In addition, upon cold exposure, some enzymes, including dehydroascorbate reductase (DHAR) and glutathione reductase (GR), for regenerating two major cellular antioxidants (ascorbate and reduced glutathione) were significantly upregulated for expression, whereas the genes encoding monodehydroascorbate reductase (MDAR) and ascorbate peroxidase-1 (APX-1) were downregulated (Table S1). These data imply that the regeneration of ascorbate, an abundant antioxidant in plants, is primarily dependent on dehydroascorbate but not monodehydroascorbate. In the ascorbate/glutathione cycle, GSH exists predominantly in the reduced form and can react chemically with ROS (singlet oxygen, superoxide, hydroxyl radicals) and a variety of toxic substrates arising from oxidative stress [31]; glutathione-S-transferase (GST) and glutathione peroxidase (GPX) catalyze the conjugation reaction. The upregulation of GST and the downregulation of GPX showed that GST plays a primary role in the detoxification of ROS.

### Osmoprotectants in *J. curcas* cold tolerance

Compatible solutes produced in response to many abiotic stresses, such as desiccation, osmotic imbalance, or low temperatures, are a heterogeneous group of small organic molecules comprised of amino acids (Ala, Gly, Pro, and Ser), polyamines, betaines, and sugars. They may act as osmoprotectants or stabilizing factors for membranes and macromolecules [52,53]. Many studies have clarified that proline drastically accumulated in plants under cold stress [54]. DGE analysis presented herein indicates that the key *J. curcas* genes involved in proline biosynthesis are upregulated upon cold exposure, and a few genes encoding enzymes, such as △^1^-pyrroline-5-carboxylate synthase (P5CS), were slightly downregulated. Similarly, the key enzyme of glycine betaine synthesis, betaine aldehyde dehydrogenase (BADH), was also upregulated for its expression, with 2.04-, 2.25-, and 1.58-fold increases after 12 h, 24 h, and 48 h of exposure to 12°C, respectively (Table S1). In addition to being an important osmotic regulator, glycine betaine may function in the stabilization of protein complexes, membranes, and transcriptional and translational machineries, and may indirectly induce H_2_O_2_-mediated signaling pathways [55].

Meanwhile, many genes involved in the accumulation of soluble sugars were also upregulated for expression to provide adequate osmoprotectants for maintaining membrane integrity and macromolecule stability against cold damage. Astonishingly, galactinol, raffinose, and/or inositol might act as the main sugars for cold resistance in *J. curcas* (instead of sucrose and trehalose that are prevalent in many other plant species) [56-58]. The genes encoding enzymes crucial for the biosynthesis of galactinol, raffinose, and inositol were significantly induced by cold (12°C) stimuli that lasted up to 48 h and were especially exemplified by the gene of galactinol synthase (GS) (upregulated 467.88-fold after 12 h at 12°C). However, similar responses were not observed for the other sugars, such as sucrose, trehalose, and stachyose (Figures 5, 6, and 7; Table S1). It has been well documented that galactinol, raffinose [58,59], stachyose, and trehalose [58,60,61] are involved in plant cold resistance, but some reports have evidenced that raffinose accumulation is not an absolute requirement for *Arabidopsis* to acquire increased cold tolerance [62]. Downregulated expression in *J. curcas* of the key enzymes in synthesizing stachyose and trehalose (downstream of raffinose, see Figure 5), such as SS, TPS, and TPP, implies that the major sugar type(s) contributing to cold tolerance may be, to a certain degree, plant-species dependent. In this regard, further quantitative measurements may be needed to determine the dominantly cold-responsive sugar factor among the three candidates (galactinol, raffinose, and inositol) in *J. curcas*.

### Cold-responsive hydrophilic proteins

In addition, some cold-responsive hydrophilic proteins, such as dehydrins and late embryogenesis abundant (LEA) proteins, may also participate in plant cold tolerance to protect the membrane and protein complexes [63-65]. Herein, the expression of the *J. curcas LEA-5* gene (CL815.Contig1_JC-CK_1A) was upregulated (at least 4.14-fold) at 12°C cold exposure across all three time points. However, a novel dehydrin gene (Unigene25029_JC-CK_1A) was found to be consistently cold repressed, thus substantiating an early report that the overexpression of a single dehydrin does not always lead to enhanced cold tolerance (e.g., overexpression of RAB18, a cold-induced dehydrin, had no effect on the cold tolerance in transgenic *Arabidopsis* plants [66]). Moreover, living organisms have to deal with the formation and stabilization of RNA secondary structures when exposed to low temperatures. Cold shock protein (CSP), a type of RNA chaperone or RNA-binding protein (RBP) with cold shock domain (CSD), played a critical role in unwinding the nucleic acid duplex and maintaining efficient translation under cold conditions [67,68]. In this study, the gene encoding the RBP protein (Unigene9159_JC-CK_1A) exhibited a 4.11-fold increase, at minimum, in expression in response to all three cold treatments (Table S1).

### Signal transduction in *J. curcas* cold stress

Presently, plant cold signal transduction pathways can be grouped into two manners of ABA-dependent and ABA-independent. Both enable plants to achieve resistance and develop adaptations to low temperatures. Under cold stress, an acute rise in cellular ABA levels induces the expression of a series of genes, most of which contain ABA-responsive *cis-*elements (e.g., ABRE, MYBR, and MYCR) in their promoters, usually represented by transcription factors of various types, such as bZIP, MYB, and MYC. The upregulated expression of the *MYB15* gene in our DGE analysis of *J. curcas* showed that ABA probably played a role in plant cold signal transduction and development of cold tolerance, which is in accordance with the findings of an earlier study on *Arabidopsis* [69]. Additionally, another cold signaling pathway mediated by CBF factors is ABA-independent and essential in triggering the cold response. CBF, with an AP2/EREBP (APETALA2/Ethylene-Responsive Element Binding Protein) domain, belongs to a small transcription factor family composed of several members, such as CBF1, CBF2, CBF3, and CBF4, that can activate the expression of downstream cold-regulated (COR) genes featured with CRT/DRE *cis-*elements (generally 5-bp core sequence of CCGAC) in the promoter regions. The expression of most CBF members (e.g., CBF1-3) cannot be induced by ABA, but are mainly modulated by its upstream regulator, ICE (Inducer of CBF expression), a bHLH transcription factor that can bind the MYC element of promoters to activate CBF expression under cold stress [70-72]. The induction of CBF1-3 in *Arabidopsis* occurred very rapidly (15 min after exposure to cold), with maximum accumulation generally attained at 3 h [73]. In contrast, expression of a *J. curcas CBF* gene (Unigene 16393_JC-CK_1A) was upregulated (1.52-fold) after 12 h of cold (12°C) exposure, and increased with increasing duration of the cold treatments (1.82- and 2.31-fold after 24 h and 48 h, respectively) (Table S1). Regardless of the different temperatures used for the cold treatments (4°C for *Arabidopsis*), the relatively slow response to cold in *J. curcas* versus *Arabidopsis* might reflect their differences in the cold-sensitivity of these two plant species with distinct terrestrial origins. Conclusively, cold exposure can trigger dramatic changes at the transcript level in plants. At present, the well elucidated cold signaling appears to be mediated by the CBF pathway. Nevertheless, microarray-based analyses indicated that only 12% of the cold responsive genes were regulated by the CBF pathway in *Arabidopsis* [44], while the majority might be ascribed to CBF beyond, such as the ABA-dependent signal transduction pathway, which remains to be understood in detail.

## Data Archiving Statement

All the clean reads have been submitted to the sequence read archive (SRA) at NCBI with accession SRR653198; This Transcriptome Shotgun Assembly (TSA) project has been deposited at GenBank/EMBL/DDBJ under the accession GAHK00000000. The version described in this paper is the first version, GAHK01000000 (GAHK01000001-GAHK01045171).

## Supporting Information

Table S1 **Selected genes of *Jatropha curcas* differentially expressed during cold stress.**
(DOC)

Table S2 **Novel genes of *Jatropha curcas* commonly expressed at three time points.**
(XLS)
