# Supplementary material for: Global Analysis of Transcriptome Responses and Gene Expression Profiles to Cold Stress of Jatropha curcas L
Source: PLoS One. 2013 Dec 9;8(12):e82817. doi: 10.1371/journal.pone.0082817 (PMC3857291; doi:10.1371/journal.pone.0082817)
Supplement: Table S1 — Selected genes of Jatropha curcas differentially expressed during cold stress. (DOC) [file pone.0082817.s001.doc]

**Table S1. Selected genes of *Jatropha curcas* differentially expressed during cold stress**

| **Category** | **Symbol** | **Enzyme (annotation in Nr)** | **Unigenes** | **Fold change** | | |
| --- | --- | --- | --- | --- | --- | --- |
|  |  |  |  | **12h** | **24h** | **48h** |
| Starch catabolism | BAM | β-amylase | Unigene804_JC-CK_1A | +30.27 | +23.26 | +10.93 |
|  | GP | Glucan phosphorylase | Unigene2449_JC-CK_1A | +4.11 | +11.39 | +3.61 |
|  | PGM | Phosphoglucomutase | Unigene5164_JC-CK_1A | +1.91 | +1.83 | +1.23 |
|  | MIPS | myo-inositol 1-phosphate synthase | Unigene680_JC-CK_1A | +1.82 | +1.91 | +3.63 |
|  | IP | myo inositol monophosphatase | Unigene4454_JC-CK_1A | +1.59 | +3.05 | +6.45 |
|  | GS | Galactinol synthase | CL2440.Contig1_JC-CK_1A | +467.88 | +191.34 | +354.59 |
|  | RS | Raffinose synthase | Unigene8178_JC-CK_1A | +5.31 | +5.06 | +1.49 |
|  | SPS | Sucrose phosphate synthase | Unigene6292_JC-CK_1A | +1.06 | +1.78 | -1.84 |
|  | SPP | Sucrose phosphate monophosphatase | Unigene4468_JC-CK_1A | -1.21 | +1.34 | +1.43 |
|  | SRE | Sucrase (Invertase) | Unigene9980_JC-CK_1A | +1.09 | -2.03 | +1.14 |
|  | UGPase | UDPG pyrophosphorylase | Unigene9304_JC-CK_1A | +1.43 | -1.30 | -1.19 |
|  | PGI | Glucose-6-phosphate isomerase | Unigene5561_JC-CK_1A | -1.55 | +1.09 | -1.36 |
|  | TPS | Trehalose-6-phosphate synthase | Unigene6824_JC-CK_1A | -1.43 | -2.10 | +1.75 |
|  | TPP | Trehalose phosphate phosphatase | Unigene15417_JC-CK_1A | -5.82 | -2.04 | -8.22 |
|  | SS | Stachyose synthase | CL5085.Contig1_JC-CK_1A | -2.71 | -3.03 | -6.92 |
| Signal transduction | PLC | Phospholipase C | Unigene2508_JC-CK_1A | +1.21 | -2.03 | -1.06 |
|  | PLDδ | Phospholipase Dδ | CL3932.Contig1_JC-CK_1A | +3.43 | +3.27 | +2.60 |
|  | CK I | Casein kinase I | Unigene8314_JC-CK_1A | +2.14 | +1.54 | +4.29 |
|  | CDPK | Calcium dependent protein kinase | CL2905.Contig1_JC-CK_1A | +5.21 | +2.95 | +2.50 |
|  | PKC | Protein kinase C | Unigene6771_JC-CK_1A | +1.49 | -2.48 | +1.27 |
|  | PP2A | Protein phosphatase 2A | CL1950.Contig2_JC-CK_1A | +2.08 | +1.51 | +3.78 |
|  | MAPK | Mitogen-activated proten kinase | Unigene1966_JC-CK_1A | +15.14 | +1.68 | +5.21 |
|  | MAPKK | MAPK-kinase | Unigene3126_JC-CK_1A | +2.57 | +1.34 | +1.84 |
|  | MAPKKK | MAPKK kinase | Unigene7859_JC-CK_1A | -1.84 | +2.04 | +3.27 |
|  | ICE1 | Inducer of CBF expression 1 | Unigene8582_JC-CK_1A | -2.58 | -1.17 | -1.71 |
|  | CBF1 | CRT/DRE binding factor | Unigene16393_JC-CK_1A | +1.52 | +1.82 | +2.31 |
|  | ADA2/3 | Transcriptional adaptor protein 2/3 | Unigene22443_JC-CK_1A | +5.39 | +5.86 | +1.01 |
|  | HOS1 | High expression of osmotic stress regulated gene 1 | CL2585.Contig1_JC-CK_1A | +44.94 | 1.00 | 1.00 |
|  | HOS2 | High expression of osmotic stress regulated gene 2 | Unigene15829_JC-CK_1A | +5.98 | +4.72 | +6.02 |
|  | HOS10 | High expression of osmotic stress regulated gene 10 | Unigene7105_JC-CK_1A | -1.49 | -1.04 | -1.49 |
|  | HOS15 | High expression of osmotic stress regulated gene 15 | Unigene12395_JC-CK_1A | +42.22 | 1.00 | +42.22 |
|  | Gcn5 | Histone acetyltransferase enzyme | Unigene6847_JC-CK_1A | +1.97 | +2.48 | -1.08 |
|  | LOS2 | Low expression of osmotically responsive gene 2 | CL3254.Contig1_JC-CK_1A | +1.29 | +1.54 | +2.35 |
|  | LOS4 | Low expression of osmotically responsive gene 4 | Unigene4045_JC-CK_1A | +1.84 | +1.61 | +1.29 |
|  | SFR6 | Sensitive to freezing 6 | Unigene8291_JC-CK_1A | -1.38 | -2.77 | +1.33 |
|  | ZAT10 | Salt-tolerance zink finger protein 10 | Unigene855_JC-CK_1A | +81.57 | +9.25 | +25.11 |
|  | ZAT12 | Salt-tolerance zink finger protein 12 | Unigene10789_JC-CK_1A | +4.92 | +1.84 | +5.03 |
|  | MYB15 | MYB transcription factor | Unigene5341_JC-CK_1A | +2.00 | +2.64 | -1.04 |
|  | SIZ1 | SUMO E3 ligase | CL1457.Contig1_JC-CK_1A | -1.38 | -62.25 | -62.25 |
| Fatty acid desaturase | SAD | △^9^-stearoyl-ACP desaturase | Unigene2223_JC-CK_1A | -1.32 | +2.07 | +3.41 |
|  | FAD2 | △^12^-Fatty acid desaturase | Unigene699_JC-CK_1A | +1.17 | +1.30 | +1.33 |
|  | ω3-FAD | ω-3-fatty acid desaturase | CL1490.Contig2_JC-CK_1A | -1.08 | +1.71 | +1.40 |
| Counterbalance of ROS | SOD | Zn-Superoxide dismutase | Unigene4592_JC-CK_1A | +1.61 | -1.30 | +1.52 |
|  | POD | Peroxidase 73 | Unigene700_JC-CK_1A | +48.50 | +6.15 | +85.63 |
|  | GPX | Glutathione peroxidase | Unigene866_JC-CK_1A | -1.17 | -2.64 | -1.54 |
|  | GST | Glutathione S-transferase | Unigene6693_JC-CK_1A | +3.23 | +1.82 | +3.18 |
|  | MDAR | Monodehydroascorbate reductase | Unigene26_JC-CK_1A | -1.57 | -1.46 | +1.12 |
|  | DHAR | Dehydroascorbate reductase | Unigene2206_JC-CK_1A | +2.46 | +2.01 | +3.01 |
|  | GR | Glutathione reductase | Unigene2439_JC-CK_1A | -1.38 | +3.10 | +1.68 |
|  | APX-1 | Ascorbate peroxidase-1 | Unigene1825_JC-CK_1A | +1.01 | -1.18 | -2.60 |
| Compatible solutes | P5CS | △^1^-pyrroline-5-carboxylate synthase | Unigene3621_JC-CK_1A | -2.45 | -2.00 | -1.28 |
|  | BADH | Betaine aldehyde dehydrogenase | Unigene3517_JC-CK_1A | +2.04 | +2.25 | +1.58 |
| Other factors | DHN | Dehydrin | Unigene25029_JC-CK_1A | -5.06 | -2.17 | -2.69 |
|  | LEA-5 | Late embryogenesis protein-5 | CL815.Contig1_JC-CK_1A | +18.51 | +4.14 | +22.94 |
|  | RBP | RNA-binding protein | Unigene9159_JC-CK_1A | +5.35 | +4.11 | +4.69 |

‘+’ indicates up-regulation, ‘-‘ indicates down-regulation.
